# Coexistence of *Legionella pneumophila* Bacteria and Free-Living Amoebae in Lakes Serving as a Cooling System of a Power Plant

**DOI:** 10.1007/s11270-014-2066-y

**Published:** 2014-07-29

**Authors:** Elżbieta Żbikowska, Hanna Kletkiewicz, Maciej Walczak, Aleksandra Burkowska

**Affiliations:** 1 Department of Invertebrate Zoology, Faculty of Biology and Environment Protection, Nicolaus Copernicus University, Lwowska 1, 87-100 Toruń, Poland; 2 Department of Animal Physiology, Faculty of Biology and Environment Protection, Nicolaus Copernicus University, Toruń, Poland; 3 Department of Environmental Microbiology and Biotechnology, Faculty of Biology and Environment Protection, Nicolaus Copernicus University, Toruń, Poland

**Keywords:** FLA, *Hartmanella* sp., *Naegleria fowleri*, *Legionella pneumophila*, Biofilm, Environment and public health, Cooling system

## Abstract

The study was aimed at determining whether potentially pathogenic free-living amoebae (FLA) and *Legionella pneumophila* can be found in lakes serving as a natural cooling system of a power plant. Water samples were collected from five lakes forming the cooling system of the power plants Pątnów and Konin (Poland). The numbers of investigated organisms were determined with the use of a very sensitive molecular method—fluorescence in situ hybridization (FISH). The result of the present study shows that thermally altered aquatic environments provide perfect conditions for the growth of *L. pneumophila* and amoebae. The bacteria were identified in the biofilm throughout the entire research period and in the subsurface water layer in July and August. *Hartmanella* sp. and/or *Naegleria fowleri* were identified in the biofilm throughout the entire research period.

## Introduction

*Legionella* spp. are Gram-negative non-encapsulated, non-endospore-forming bacilli, ranging in size from 0.3–0.9 μm × 2–20 μm, with a single, polar flagellum. First identified in 1976, *Legionella* spp. (Saint and Ho 1999; Gomez-Valero et al. 2009; Huang and Hsu 2010) are one of the main groups of pathogenic bacteria transmitted via water (Fields et al. 2002). Human infection by *Legionella pneumophila* usually results from inhaling aerosol—droplets of water which contain bacterial cells (Pancer et al. 2008; Declerck 2010). In Europe, *L. pneumophila* are responsible for 95 % of all reported cases of legionellosis, the remaining cases being caused by *Legionella longbeachae* (Whiley and Bentham 2011).

Although *Legionella* can multiply in temperatures ranging from 20 to 40 °C (Grabińska-Łoniewska 2010; Diederen 2008), the optimum temperature for their growth is 32–35 °C. However, they can survive in an aquatic environment in temperatures ranging from 0 to 68 °C and with pH ranging from 5.0 to 8.5 (Diederen 2008). They need aerobic conditions, oxygen concentration being another determinant of their growth. *L. pneumophila* are known to thrive in biofilms which form at the solid–liquid or at liquid–air interface (floating biofilm, surface microlayer) (Flemming et al. 2000). The natural habitat of *Legionella* spp*.* is the aquatic environment: they are found both in natural water bodies (lakes, ponds, rivers, thermal waters) and in man-made (anthropogenic) ones (swimming pools, water supply systems, cooling towers) (Huang and Hsu 2010). They have also been recovered from compost, sewage, soils (Devos et al. 2005), and saline water (Żbikowska et al. 2013; Walczak et al. 2013).

The number of these microorganisms is usually higher in man-made ecosystems providing perfect conditions for their growth (Guyard and Low 2011) than in natural ecosystems, where their number is usually low (Steinert et al. 2002). The growth and survival rate of *Legionella* in the environment depend mainly on their ability to develop symbiotic relationships with protists. *Legionella* have been identified inside several ciliates including *Tetrahymena* and *Cyclidium* species as well as inside amoeba species belonging to *Acanthamoeba*, *Hartmanella*, *Valkampfia*, and *Naegleria* genera (Lee and West 1991; Paszko-Kolva et al. 1993; States et al. 1989; Kramer and Ford 1994; Henke and Seidel 1986; Fields 1996; Vandenesch et al. 1990), referred to as free-living amoebae (FLA), ubiquitous and opportunistic protists, which can induce human and animal diseases (Dendana et al. 2008).

One FLA species in particular, *Naegleria fowleri*, is identified as the causative agent of primary amoebic meningoencephalitis (PAM), an acute, rapidly fatal disease of the central nervous system that is observed in young people after exposure to contaminated water in public swimming pools or lakes (Martinez 1985). In the majority of the reported cases, the invasion occurred in freshwater bodies in swimmers or divers. The infection develops after the amoeba enters the nasal passage, then penetrates through the ethmoid bone and travels to the brain (Fiordalisi et al. 1992), after which the victim dies within several days. Although PAM is not a common disease, an increase in the number of the recorded cases has been observed since the 1990s. (De Napoli et al. 1996; Taylor et al. 1996; Marciano-Cabral et al. 2003; Gyori 2003; Okuda et al. 2004; Schuster and Visvesvara 2004; Craun et al. 2005). Moreover, accelerated multiplication of *Naegleria* was observed in the presence of several strains of *Enterobacteriaceae* (Kyle and Noblet 1985; Singh and Dutta 1984). Thermally altered environments, for example lakes altered by power plants, provide highly favorable conditions for the growth of thermophilic *Naegleria* and thermophilic bacteria, the latter being a vital element of amoebae diet (Tyndall et al. 1989; Sykora et al 1983). These environments (lakes) often are used by people for recreation (swimming, surfing). Both FLA and thermophilic bacteria can thrive in thermally polluted water bodies including water systems used by industries. Taking into consideration that these two groups of thermophilic organisms include potential pathogens of humans and animals, regular monitoring of thermally altered water bodies should be a crucial part of environmental diagnostics. Data on the spread of *N. fowleri* in the environment are collected mainly in the USA (Stevens et al., 1977; John and Howard 1995), Australia (Mackowiak et al. 2010), New Zealand (Brown et al. 1983), and Asia (Shin and Im 2004), where natural thermal water conditions promote the expansion of the species. In Europe, despite the alarming statistics (17 fatal cases of PAM in Ústí nad Labem in the years 1962–1965, Červa and Novák 1968), the problem of deadly amoebae in heated waters was abandoned, probably due to the inefficient diagnostic techniques.

*N. fowleri* is the only potentially pathogenic species out of nearly 30 belonging to the *Naegleria* genus (Martinez 1985; Visvesvara et al. 2007). Due to several morphological similarities, non-specific behavior in a culture, as well as a typical antigenic structure, *N. fowleri* is difficult to differentiate from other thermotolerant species, particularly from non-pathogenic *Naegleria lovaniensis* (Reveiller et al. 2002). The introduction of molecular diagnostic techniques significantly increased chances of obtaining reliable data on the spread of the pathogenic amoebae in the environment (Pélandakis and Pernin 2002; Reveiller et al. 2002; Marciano-Cabral et al. 2003).

The present paper is aimed at filling gaps in the knowledge of the distribution of thermophilic, potentially pathogenic FLA and coexisting *Legionella* sp. in the biofilm of thermally polluted lakes serving as a cooling system of a power plant located in central Europe (Poland). The biofilm formed on the liquid–air interface was monitored for the presence of thermophilic pathogens due to the regular presence of Gram-negative bacteria including *Legionella* sp*.* and *L. pneumophila*. Bacteria in this formation build a mucopolysaccharide matrix, enabling them to colonize free-living amoebae.

## Materials and Methods

**Fig. 1:**
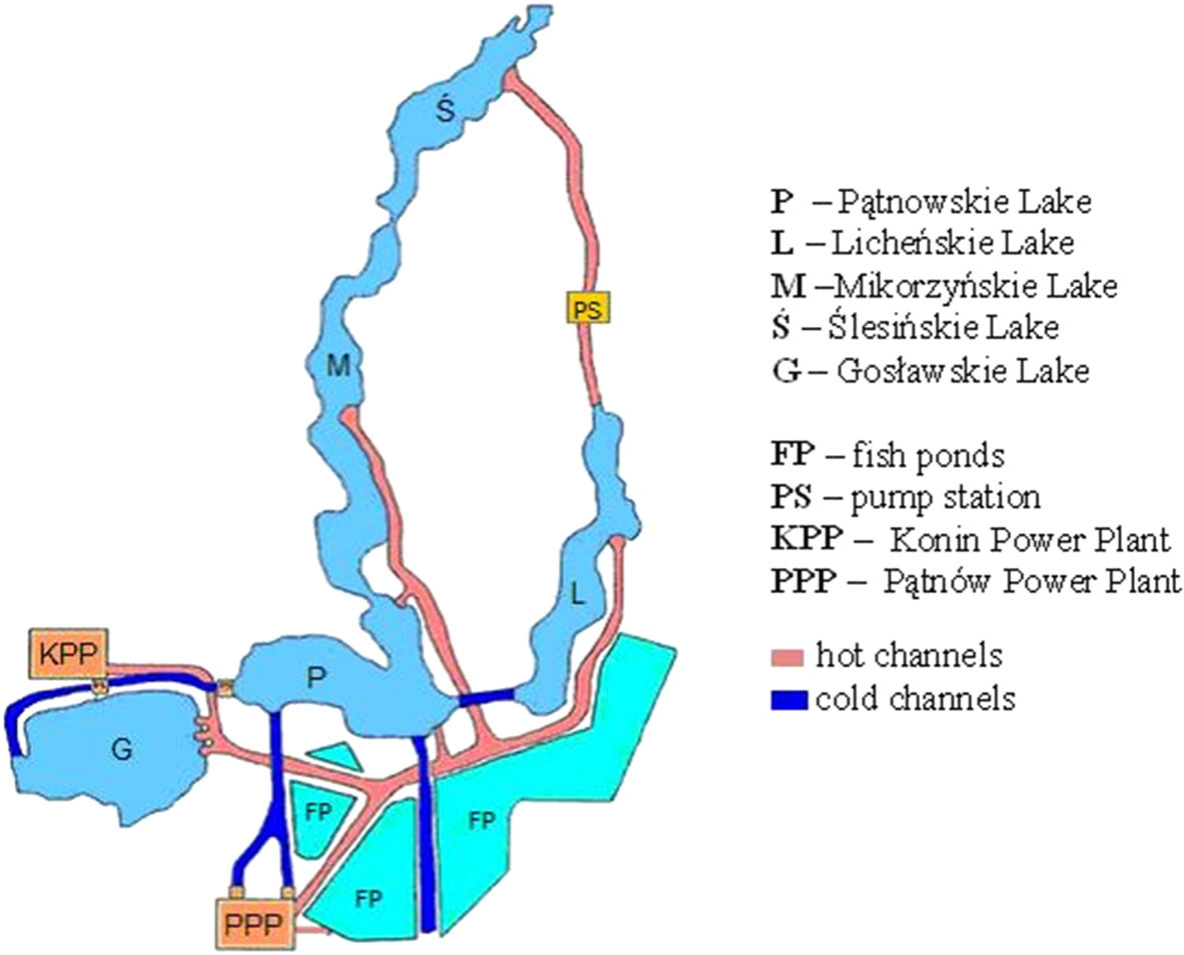
The cooling system of the power plants Pątnów and Konin

Water samples were collected from five lakes forming the cooling system of the power plants Konin (founded in 1958 and its electric power is 198 MW) and Pątnów (founded in 1967 and its electric power is 200 MW). The heated water from the Konin power plant is discharged to lakes Gocławskie, Pątnowskie, and Mikorzyńskie. From the Pątnów power plant, the heated water is discharged to lakes Pątnowskie, Mikorzyńskie, and Licheńskie. From Licheńskie Lake, the water flows to Ślesińskie Lake (large loop). While flowing, the water cools and is taken by the power plants. Joined with a system of canals, the lakes have a total length of 26 km. The lake–canal system is a closed loop (in terms of the power plant operation), where the water flow is regulated by water culverts and pumping stations. Water from the lakes cools the power generation units of both power plants and returns to the lakes heated (Fig. 1).

All five lakes are used for recreation (swimming, surfing). Above lakes Mikorzyn, Ślesin, and Pątnów are mainly holiday resorts. These lakes are also centers of sailings. Lake Gocławskie is mainly used as a reservoir for surfing, and Lichen Lake is used for recreation, swimming, and fishing.

The temperature of the water discharged from the power plant is on average between 6.0 and 9.0 °C higher than the temperature of the water collected from the lakes. This has led to the permanent increase in the water temperature, ranging between 26 and 31 °C in summer. All the lakes have aerobic conditions in the epilimnion layer.

Water samples were collected from all five lakes in May, July, August, and October. Each sampling procedure involved collecting water from the biofilm, 1 l (the surface microlayer to the maximum depth of 150 μm), using a plexiglass plate and from a depth of about 20 cm, 1 l, using a sterile pipette. Samples were not collected from sediments because of anoxic conditions. Water samples were transferred to sterile glass bottles and then transported to the laboratory at 7 °C.

The following physicochemical parameters of water were measured: temperature, pH value (with the ELMETRON pH meter), and oxygen saturation (with the HANNA Instruments oximeter). The numbers of bacteria belonging to *Legionella* sp*.* and *L. pneumophila* and the investigated eukaryotic organisms (*Naegleria* sp., *N. fowleri*, and *Hartmanella* sp.) were determined with the use of the molecular fluorescence in situ hybridization (FISH) method.

In the laboratory, water samples from each lake were averaged, then fixed with formamide and filtered through polycarbonate membrane filters with a 0.22-μm pore size in order to capture particles bigger than 0.22 μm. The hybridization was performed according to Grimm et al. (2001). Bacterial cells retained on the surface of the membrane were hybridized using *Legionella*-specific fluorescence-labelled (with dye CY3) oligonucleotide probes LEG705 and LEGPNE1 and fluorescein-labelled eukaryote-specific probes HART498, NAEG1088, and NAE1041. The probes were suspended in the hybridization buffer consisting of formamide (whose concentration depended on the probe sequence [vol/vol]; NaCl 0.9 mM, sodium dodecyl sulfate 0.01 %, Tris/HCl, pH 7.6, 20 mM). This solution was applied to the surface of the filter with captured cells. For probes LEG705 and LEGPNE1, the formamide concentration was 25 %, and for probes NAEG1088 and NAE1041, the formamide concentration was 30 %; for probe HART498, the formamide concentration was 40 %. The probe concentration in the hybridization buffer was 30 ng of probes for prokaryotic organisms and 150 ng of probes for eukaryotic organisms.

The filters were then placed for 2 h in a hybridization chamber and in an ultrathermostat at 46 °C. After that, in order to remove the unbound probes, the filters were placed for 15 min in the washing buffer with a temperature of 48 °C (the composition of the buffer—20 mM Tris/HCl, pH 7.6; 0.01 % sodium dodecyl sulfate; 5 mM EDTA; and 160 mM NaCl for probes LEG705 and LEGPNE1; 5 mM EDTA and 56 mM NaCl for probe HART498; 5 mM EDTA and 112 mM NaCl for probes NAEG1088 and NAE1041), rinsed with distilled water, and dried.

Subsequently, the filters were covered with a mixture of immersion oil and Citifluor AF2 (Citifluor Ltd., London, UK). Fluorescence was detected using an Olympus BX50 epifluorescence microscope with a 50-W mercury high-pressure bulb and the appropriate filter set 00 and 10. The slides with hybridized prokaryotic cells were analyzed at a total of ×1,000 magnification while the slides with eukaryotic cells were analyzed at ×100 and ×400 magnifications. Color micrographs were taken with digital image processing (Olympus XC50) using the software package Cell^B^ v. 3.1.. The number of bacteria in the investigated slides was evaluated using the MultiScan Base program.

The sensitivity of the method was controlled with respect to *L. pneumophila* and eukaryotic cells. To this end, a cell suspension of a known number of cells (10^3^ cm^−3^) was prepared. Then, 1 ml of the cell suspension was filtered with a membrane filter and the procedure of FISH was performed. After estimation of the number of hybridized cells, the sensitivity of the method was determined.

The tests involved the evaluation of the morphological features of hybridized amoebae with special emphasis on the size and other features typical of the *Naegleria* and *Hartmanella* genera. *Naegleria* spp. are uniform in shape—a cell is 10–15 μm. The cytoplasm is slightly granular and has a clearly visible bright halo with a dense nucleus. Numerous vacuoles are usually seen in the cytoplasm. Trophozoites move by extending and contracting their rounded pseudopodium (lobopodium), bright on the edges and filled with granular cytoplasm. The posterior end of the cell, a hyaline uroid, has many small pseudopodia.

The trophozoites of *Hartmanella* reach the size of 25–40 μm, have an elongated shape, and produce monopodial lobopodia. The cytoplasm has numerous bright areas, corresponding to vacuolar vesicles. Due to the morphological similarity of pathogenic *Naegleria* and *Hartmanella* to non-pathogenic species and due to a slight risk of non-specific binding of the probes to the particles of organic matter after hybridization, the combination of two methods, i.e., molecular (FISH) and morphological, can provide an accurate evaluation of the studied FLA.

The number of amoebae identified by fluorescence in situ hybridization and confirmed in the morphological analysis was recalculated per 1 dm^3^ of the sampled water with the use of the abovementioned formula.

Selecting *N. fowleri* for the research was imposed by their highly pathogenic impact on humans and animals. Selecting *Hartmanella* was based on the suggestion of Brieland et al. (1997) that the passage of *L. pneumophila* through *Hartmanella* seemed to increase the pathogenicity of the bacteria for humans.

Statistical analyses were conducted using program STATISTICA 6.0. Analysis of variance (ANOVA) and correlation were the statistical methods used in calculations. The results were analyzed considering the presence of all investigated phylogenetic bacterial (*Legionella* sp. and *L. pneumophila*) and eucaryotic (*Naegleria* sp., *N. fowleri*, *Hartmanella* sp.) groups in relation to the physicochemical parameters of water in the investigated samples of water and the season of the year.

## Results

### Physicochemical Parameters

Physicochemical parameters of the collected water are shown in Table 1. During the research period, the average water temperature ranged from 17.66 °C in October to 27.98 °C in July. The fluctuations in water temperatures were considered significant (*p* < 0.01). Water in all investigated lakes was alkaline (Table 1). In May, July, and August, slight differences in water pH were observed. In October, water pH had the lowest value (7.84). All the investigated lakes and canals had aerobic conditions. The highest oxygen concentration was noted in May while a significantly lower oxygen concentration was noted in July and August. Seasonal fluctuations in water oxygenation were statistically significant (*p* < 0.05) (Table 1).

**Table 1:** The physicochemical parameters of the collected water

Month		Temperature [°C]		pH		O_2_ [%]		O_2_ [ppm]	
		Mean	SD	Mean	SD	Mean	SD	Mean	SD
Lakes	May	20.84	3.00	8.50	0.10	167.80	61.52	14.36	6.13
	July	27.98	3.92	8.42	0.20	78.60	21.38	6.18	2.31
	August	23.08	1.96	8.40	0.12	83.20	8.83	6.94	0.80
	October	17.66	1.07	7.84	0.50	96.50	31.29	8.65	3.01
Canals	July	30.35		8.15		71.00		5.10	

### *Legionella* in the Biofilm and in the Subsurface Water Layer

**Fig. 2:**
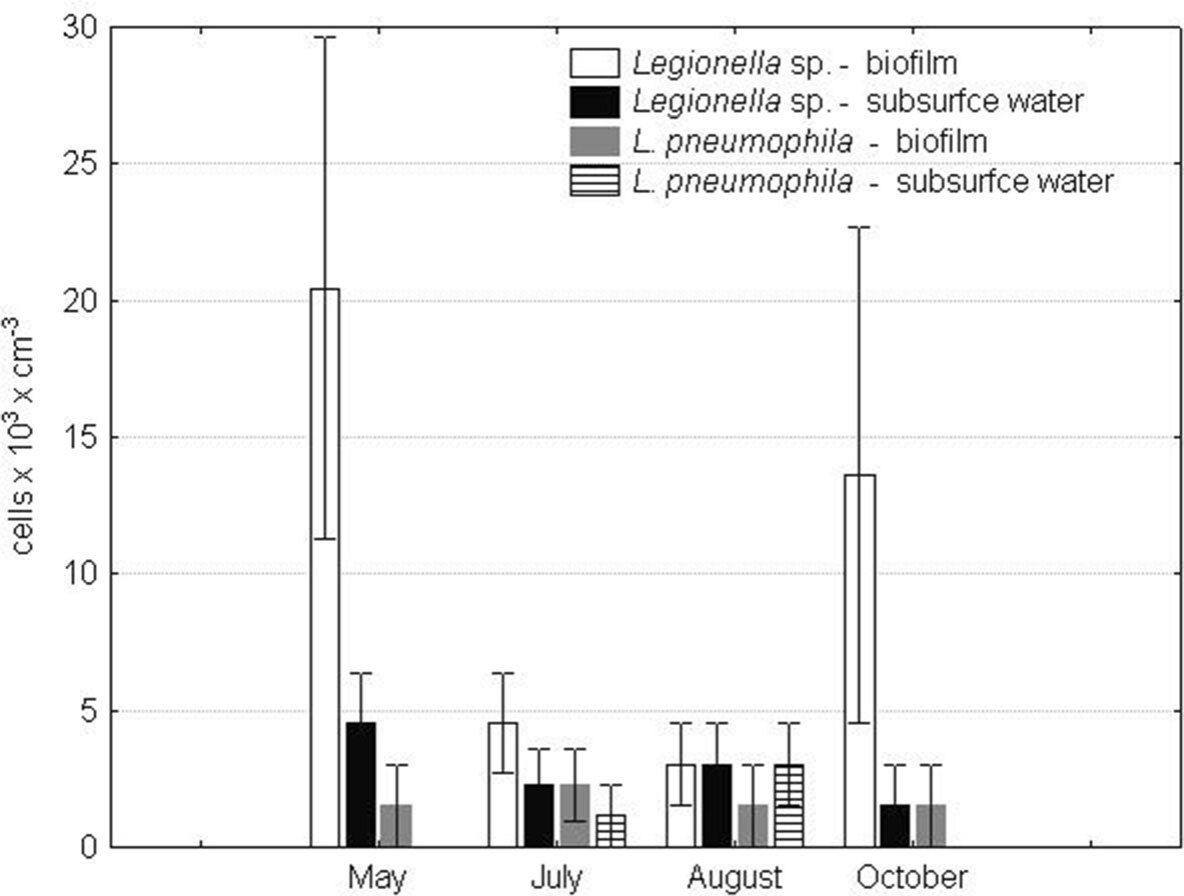
The numbers of *Legionella* sp. and *L. pneumophila* in lakes serving as a cooling system in a power plant

Bacteria of the *Legionella* genus were identified in the collected water samples throughout the entire research period (Fig. 2) The highest numbers of *Legionella* sp*.* in the biofilm were noted in May and October, 20.43 × 10^3^ and 13.62 × 10^3^ cells cm^−3^, respectively. Substantially lower numbers of *Legionella* sp. were noted in July (4.54 × 10^3^ cells cm^−3^) and August (3.02 × 10^3^ cells cm^−3^). In the subsurface water layer, the highest number of *Legionella* sp. was noted in May (4.54 × 10^3^ cells cm^−3^). In July and August, the number decreased by half to further decrease in October when it was 1.51 × 10^3^ cells cm^−3^.

**Fig. 3:**
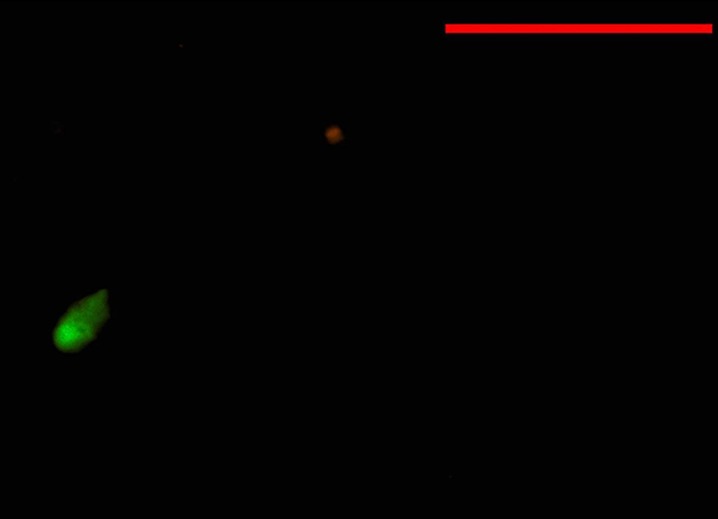
Positive result of FISH hybridization for *Naegleria fowleri*; *bar* = 50 mm

*L. pneumophila* were identified in the biofilm throughout the entire research period; the highest number was noted in July at 2.27 × 10^3^ cells cm^−3^. Half of this number was noted in May and October, at 1.51 × 10^3^ cells cm^−3^ (Fig. 3). In the subsurface water layer, *L. pneumophila* were identified only in July (1.13 × 10^3^ cells cm^−3^) and August (3.02 × 10^3^ cells cm^−3^). In May, July, and October, *L. pneumophila* were more abundant in the biofilm. In August, they were more abundant in the subsurface water layer. The number of *L. pneumophila* in the biofilm was positively correlated with water temperature (*r* = 0.86).

### FLA in the Biofilm and in the Subsurface Water Layer

*Naegleria* sp., *N. fowleri*, and *Hartmanella* sp. were identified in the water samples collected from the lakes. Pathogenic *N. fowleri* (Fig. 3) were identified in the biofilm in summer, at 54 cells cm^−3^ in July and 57.2 cells cm^−3^ in August (Table 2). They were also identified in the subsurface water layer in May and August, at 54.4 and 1.6 cells cm^−3^, respectively.

**Table 2:** The numbers of FLA (cells cm^−3^) in lakes serving as a cooling system in a power plant

Month		*Naegleria* sp.	*Naegleria fowleri*	*Hartmanella*
May	Biofilm	0	0	51.9
	Subsurface water	52.8	54.4	53.5
July	Biofilm	0	53.9	55.1
	Subsurface water	0	0	0
August	Biofilm	0	57.2	0
	Subsurface water	0	1.6	0
October	Biofilm	0	0	54.7
	Subsurface water	0	0	0

**Fig. 4:**
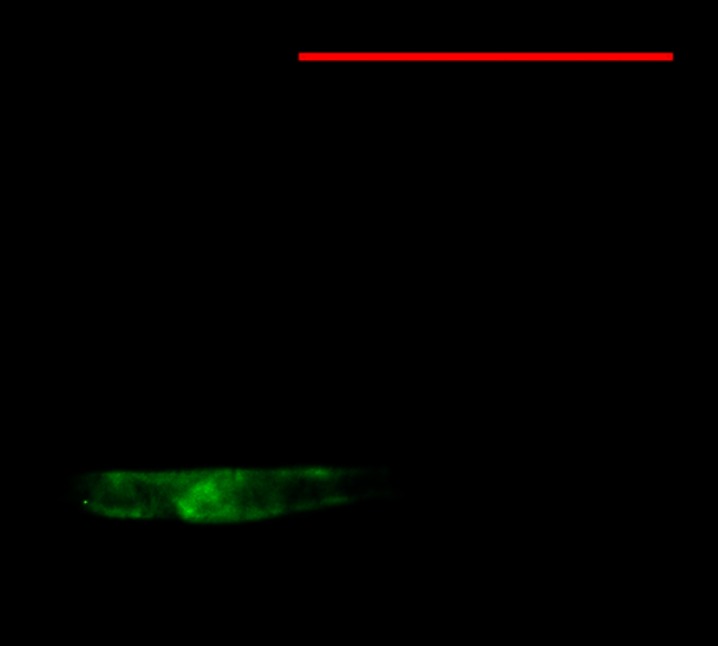
Positive result of FISH hybridization for *Hartmanella*; *bar* = 50 mm

Amoebae of the *Hartmanella* genus were more frequently recorded in the biofilm than in the subsurface water layer; in the former, they were identified in May, July, and October and the recorded number was always 54.4 cells cm^−3^ (Table 2). In the latter, they were identified only in May (54.4 cells cm^−3^). *Hartmanella* (Fig. 4) were not identified in any of the samples collected from the discharge canals.

*Naegleria* spp. were not identified in the biofilm throughout the entire research period. In the subsurface water layer, they were identified only in May (54.4 cells cm^−3^). In the subsurface water layer, the number of all investigated FLA was positively correlated with water oxygenation (*r* = 0.96) and with the number of *Legionella* (*r* ranging from 0.88 to 0.90).

## Discussion

Common in aquatic environments, bacteria belonging to the *Legionella* genus have been identified in natural and man-made (anthropogenic) water bodies (Diederen 2008), in the biofilm and in the subsurface water layer. Their growth rate depends on several chemical and physical factors including temperature, water pH, and the concentration of compounds dissolved or suspended in water, as well as on biological factors including the presence of several groups of Eubacteria, Cyanophyta, and Protista.

Known to play a fundamental role in the growth of *L. pneumophila*, water temperature and oxygenation were subject to the most considerable seasonal fluctuations in the investigated lakes (the cooling system of a power plant). In addition, previous studies indicate that a higher temperature intensifies the mineralization of organic matter and increases the growth rate of bacteria (Zdanowski 1998), including *L. pneumophila* (Devos et al. 2005; Declerck 2010).

The results of the microbiological tests indicate that bacteria belonging to the *Legionella* genus were common in the investigated lakes and canals, with their highest number recorded in May. According to Świątecki and Zdanowski (2007), in May, massive algal and cyanobacterial blooms can be expected. What is more, the results of Declerck’s research (Declerck 2010) suggest that photosynthetic cyanobacteria are able to promote the growth of *Legionella*. However, despite the high number of *Legionella*, in spring, the number of *L. pneumophila* was rather low and they were identified only in the biofilm. This may be related to the low water temperature (in May, 21 °C on average); although the temperature range for the growth of *L. pneumophila* falls between 20 and 40 °C (Grabińska-Łoniewska 2010; Diederen, 2008), the temperature ensuring their optimal growth rate ranges from 32 to 35 °C.

In July, despite the significantly lower number of *Legionella* sp., the number of *L. pneumophila* increased, which may be attributed to the considerable rise in water temperature (29 °C on average). Owing to favorable conditions, water in the discharge canals also contained high numbers of *L. pneumophila.* High water temperature (above 28 °C), good aerobic conditions, and high number of Eubacteria contributed tremendously to the growth of these bacteria.

In August, the populations of *Legionella* sp. and *L. pneumophila* in the biofilm decreased slightly as a result of deficiency or exhaustion of easily digestible organic matter (Zdanowski 1998). However, Devos et al. (2005) suggest that the decrease may be related to the emergence of bacteria that inhibit the growth of *Legionella*. According to Toze et al. (1990), 32 % of heterotrophic bacteria including *Pseudomonas* sp. and *Aeromonas* sp. can inhibit the growth of *Legionella* sp. Similar results documenting the decrease in the number of *Legionella* sp. and *L. pneumophila* in late summer were obtained by Declerck et al. (2007a).

During autumn, in the biofilm, the number of *Legionella* sp. and *L. pneumophila* increased in the fall despite the temperature drop (13.62 × 10^3^ and 1.51 × 10^3^ cells cm^−3^, respectively). In view of the fact that, according to Devos et al. (2005), *Legionella* can obtain essential nutrients from decaying organic matter, the observed accumulation of particulate organic matter in the biofilm may have increased the number of these bacteria. In addition, as has been reported in numerous studies, the accumulation of organic matter and the subsequent rapid growth of bacteria can be observed in the biofilm (Walczak and Swiontek-Brzezinska 2010; Burkowska et al. 2010). By contrast, the number of *L. pneumophila* in the subsurface water layer was very low. This fact can be attributed to different conditions in this microenvironment (no biofilm formation).

*L. pneumophila* were identified in the biofilm throughout the entire research period. Their presence in natural water bodies is correlated with the presence of other microorganisms, a valuable source of exogenous amino acids.

Numerous studies indicate that the growth of *L. pneumophila* in aquatic environments (along with biofilm) is promoted by three microbial groups, i.e., amoebae, cyanobacteria, and some heterotrophic bacteria (Devos et al. 2005; Declerck 2010) including *Flavobacterium* sp*.*, *Alcaligenes* sp*.*, and *Acinetobacter* sp*.* All the heterotrophic bacteria species were previously identified in the biofilm by Donderski et al. (1999) and Kalwasińska and Donderski (2005). Moreover, the survival rate of *L. pneumophila* in biofilm may be enhanced by the algae *Scenedesmus* spp., *Chlorella* spp., and *Gleocystis* spp. as well as by free-living amoebae, which, according to Loret and Greub (2010), are commonly found at the edges of biofilms, where they can feed on ample amounts of algae, fungi, and bacteria.

In the investigated lakes, *Hartmanella* sp. and *N. fowleri* were identified in the biofilm throughout the entire research period (Table 2). In the symbiotic relationship between pathogenic strains of *Legionella* and FLA described by Greub and Raoult (2003), Declerck et al. (2007b), Hsu et al. (2009), Buse and Ashbolt (2011), and others, FLA protect the bacteria from unfavorable environmental conditions (Borella et al. 2005; Marciano-Cabral et al. 2010) while rapidly multiplying bacteria provide FLA with nutrients (Greub and Raoult 2004).

In the anthropogenically altered environments, the symbiotic relationship between pathogenic amoebae (particularly *N. fowleri*) and pathogenic bacteria poses a serious health risk for humans. The investigated lakes serving as a cooling system of a power plant are also a recreational area. The solid (floating) and dispersed (aerosol) biofilms containing pathogens pose a serious risk for swimmers and amateurs of water sports (Goutziana et al. 2008). The risk is increased by the fact that the growth of *N. fowleri* in thermally polluted aquatic environments in colder regions of the world is not naturally regulated by other thermophilic microorganisms competing with pathogenic FLA (Marciano-Cabral 1988).

The number of pathogenic amoebae in the collected water samples ranged from 0.0 to 57.2 cells cm^−3^, the values which can be perceived as a real threat to human health. Although a dangerous level of *N. fowleri* was not determined for the freshwater bodies, Cabanes et al. (2001) using mouse models for experimental inoculation assessed that the probability of infection in swimmers is 8.5 × 10^−8^ with an amoebae concentration of 100 cells dm^−3^. However, even a lower concentration of amoebae (25 cells dm^−3^) was responsible for seven cases of infection in humans in Florida, USA (Wellings et al. 1977). A dangerous infective level of *L. pneumophila* is considered to be 1 × 10^3^ cells cm^−3^, which is capable of growing on culture media. The results of the present research indicate a higher level of these bacteria in the investigated lakes. However, the research was based on the molecular FISH method, used for detection of both culturable and non-culturable cells. The prior validation of the method indicates that its sensitivity in this case is about 100 to 1,000 times higher than the sensitivity of the culture method, which therefore implies that the concentration of *L. pneumophila* did not exceed the invasive level.

## Conclusions

In temperate climates, natural but thermally altered aquatic environments provide perfect conditions for the growth of *L. pneumophila* and potentially pathogenic amoebae. The fact that the concentration of both *L. pneumophila* and FLA was higher in the biofilm than in the subsurface water layer indicates an increased risk of the invasion of pathogenic microorganisms in amateurs of water sports. Since seasonal fluctuations in the number of these organisms in the biofilm are directly connected with a temperature rise (the highest temperatures recorded in the peak of the summer season), monitoring freshwater bodies for the presence of these pathogens seems of the utmost importance.
